# Prognostic value of automated KI67 scoring in breast cancer: a centralised evaluation of 8088 patients from 10 study groups

**DOI:** 10.1186/s13058-016-0765-6

**Published:** 2016-10-18

**Authors:** Mustapha Abubakar, Nick Orr, Frances Daley, Penny Coulson, H. Raza Ali, Fiona Blows, Javier Benitez, Roger Milne, Herman Brenner, Christa Stegmaier, Arto Mannermaa, Jenny Chang-Claude, Anja Rudolph, Peter Sinn, Fergus J. Couch, Peter Devilee, Rob A. E. M. Tollenaar, Caroline Seynaeve, Jonine Figueroa, Mark E. Sherman, Jolanta Lissowska, Stephen Hewitt, Diana Eccles, Maartje J. Hooning, Antoinette Hollestelle, John W. M. Martens, Carolien H. M. van Deurzen, kConFab Investigators, Manjeet K. Bolla, Qin Wang, Michael Jones, Minouk Schoemaker, Jelle Wesseling, Flora E. van Leeuwen, Laura Van ‘t Veer, Douglas Easton, Anthony J. Swerdlow, Mitch Dowsett, Paul D. Pharoah, Marjanka K. Schmidt, Montserrat Garcia-Closas

**Affiliations:** 1 Division of Genetics and Epidemiology, The Institute of Cancer Research, 15 Cotswold Road, Sutton, London, SM2 5NG UK; 2 Breast Cancer Now Toby Robins Research Centre, Division of Breast Cancer Research, The Institute of Cancer Research, London, UK; 3 Cancer Research UK Cambridge Institute, University of Cambridge, Cambridge, UK; 4 Centre for Cancer Genetic Epidemiology, Department of Oncology, University of Cambridge, Cambridge, UK; 5 Human Genetics Group, Human Cancer Genetics Program, Spanish National Cancer Research Centre (CNIO), Madrid, Spain; 6 Centro de Investigacion en Red de Enfermedades Raras (CIBERER), Valencia, Spain; 7 Cancer Epidemiology Centre, Cancer Council Victoria, Melbourne, Australia; 8 Centre for Epidemiology and Biostatistics, Melbourne School of Population and Global health, The University of Melbourne, Melbourne, Australia; 9 Division of Clinical Epidemiology and Aging Research, German Cancer Research Center (DKFZ), Heidelberg, Germany; 10 Division of Preventive Oncology, German Cancer Research Center (DKFZ) and National Center for Tumor Diseases (NCT), Heidelberg, Germany; 11 German Cancer Consortium (DKTK), German Cancer Research Center (DKFZ), Heidelberg, Germany; 12 Saarland Cancer Registry, Saarland, Germany; 13 School of Medicine, Institute of Clinical Medicine, Pathology and Forensic Medicine, Cancer Center of Eastern Finland, University of Eastern Finland, Kuopio, Finland; 14 Imaging Center, Department of Clinical Pathology, Kuopio University Hospital, Kuopio, Finland; 15 Division of Cancer Epidemiology, German Cancer Research Center (DKFZ), Heidelberg, Germany; 16 University Cancer Center Hamburg, University Medical Center Hamburg-Eppendorf, Hamburg, Germany; 17 Department of Pathology, Institute of Pathology, Heidelberg University Hospital, Heidelberg, Germany; 18 Department of Laboratory Medicine and Pathology, Mayo Clinic, Rochester, MN USA; 19 Department of Human Genetics and Department of Pathology, Leiden University Medical Center, Leiden, The Netherlands; 20 Department of Surgery, Leiden University Medical Center, Leiden, The Netherlands; 21 Department of Medical Oncology, Family Cancer Clinic, Erasmus MC Cancer Institute, Rotterdam, The Netherlands; 22 Usher Institute of Population Health Sciences and Informatics, The University of Edinburgh, Edinburgh, UK; 23 Divisions of Cancer Epidemiology and Genetics, National Cancer Institute, Rockville, MD USA; 24 Department of Cancer Epidemiology and Prevention, M. Sklodowska-Curie Memorial Cancer Center and Institute of Oncology, Warsaw, Poland; 25 Laboratory of Pathology, National Cancer Institute, National Institutes of Health, Rockville, MD USA; 26 Faculty of Medicine Academic Unit of Cancer Sciences, Southampton General Hospital, Southampton, UK; 27 Family Cancer Clinic, Department of Medical Oncology, Erasmus MC Cancer Institute, Rotterdam, The Netherlands; 28 Department of Pathology, Erasmus MC Cancer Institute, Rotterdam, The Netherlands; 29 Department of Genetics, QIMR Berghofer Medical Research Institute, Brisbane, Australia; 30 Centre for Cancer Genetic Epidemiology, Department of Public Health and Primary Care, University of Cambridge, Cambridge, UK; 31 Division of Molecular Pathology, Netherlands Cancer Institute, Antoni van Leeuwenhoek Hospital, Amsterdam, The Netherlands; 32 Division of Psychosocial Research and Epidemiology, Netherlands Cancer Institute, Antoni van Leeuwenhoek Hospital, Amsterdam, The Netherlands; 33 Division of Breast Cancer Research, The Institute of Cancer Research, London, UK; 34 Academic Department of Biochemistry, Royal Marsden Hospital, Fulham Road, London, UK

**Keywords:** Breast cancer, Automated KI67, Visual KI67, Prognostication

## Abstract

**Background:**

The value of KI67 in breast cancer prognostication has been questioned due to concerns on the analytical validity of visual KI67 assessment and methodological limitations of published studies. Here, we investigate the prognostic value of automated KI67 scoring in a large, multicentre study, and compare this with pathologists’ visual scores available in a subset of patients.

**Methods:**

We utilised 143 tissue microarrays containing 15,313 tumour tissue cores from 8088 breast cancer patients in 10 collaborating studies. A total of 1401 deaths occurred during a median follow-up of 7.5 years. Centralised KI67 assessment was performed using an automated scoring protocol. The relationship of KI67 levels with 10-year breast cancer specific survival (BCSS) was investigated using Kaplan–Meier survival curves and Cox proportional hazard regression models adjusted for known prognostic factors.

**Results:**

Patients in the highest quartile of KI67 (>12 % positive KI67 cells) had a worse 10-year BCSS than patients in the lower three quartiles. This association was statistically significant for ER-positive patients (hazard ratio (HR) (95 % CI) at baseline = 1.96 (1.31–2.93); *P* = 0.001) but not for ER-negative patients (1.23 (0.86–1.77); *P* = 0.248) (*P*-heterogeneity = 0.064). In spite of differences in characteristics of the study populations, the estimates of HR were consistent across all studies (*P*-heterogeneity = 0.941 for ER-positive and *P*-heterogeneity = 0.866 for ER-negative). Among ER-positive cancers, KI67 was associated with worse prognosis in both node-negative (2.47 (1.16–5.27)) and node-positive (1.74 (1.05–2.86)) tumours (*P*-heterogeneity = 0.671). Further classification according to ER, PR and HER2 showed statistically significant associations with prognosis among hormone receptor-positive patients regardless of HER2 status (*P*-heterogeneity = 0.270) and among triple-negative patients (1.70 (1.02–2.84)). Model fit parameters were similar for visual and automated measures of KI67 in a subset of 2440 patients with information from both sources.

**Conclusions:**

Findings from this large-scale multicentre analysis with centrally generated automated KI67 scores show strong evidence in support of a prognostic value for automated KI67 scoring in breast cancer. Given the advantages of automated scoring in terms of its potential for standardisation, reproducibility and throughput, automated methods appear to be promising alternatives to visual scoring for KI67 assessment.

**Electronic supplementary material:**

The online version of this article (doi:10.1186/s13058-016-0765-6) contains supplementary material, which is available to authorized users.

## Background

Despite endorsements by several international guidelines [1, 2] KI67 is yet to gain widespread application as a prognostic and/or predictive marker in breast cancer [3]. This is due, largely, to methodological variability in KI67 scoring (such as antibody type, specimen type, type of fixative, antigen retrieval methods, method of scoring, etc.), and limitations in the design and analyses of studies that have reported on this marker [3–7].

In the majority of settings, KI67 is evaluated visually by a pathologist even though there is yet to be consensus regarding which regions to score between the invasive edge, hot spots or the entire spectrum of the whole section or tumour core [8]. As a result, both the intra-observer and, especially, the inter-observer reproducibility of visually derived KI67 scores have been shown to be poor [9–11]. This has not only hampered inter-study comparability for KI67, but has fuelled concerns regarding its analytical validity [3]. To address some of the methodological issues related to KI67 assessment, the International KI67 in Breast Cancer Working Group published recommendations aimed at the standardisation of the analytical processes for KI67 evaluation [8]. This panel, however, fell short of making recommendations regarding the preferred method of scoring for KI67 between visual and automated. Several reports suggest that automated methods could address some of the problems associated with visual scoring [11–19]. These methods are high throughput and are not limited by intra-observer variability. However, concerns exist regarding the accuracy of automated methods and the prognostic power of KI67 derived using these methods relative to that derived visually by pathologists. Few relatively small studies have reported a head-to-head comparison between scores derived using both methods in terms of prognostic properties, and the results from these are conflicting [11, 17–19].

The majority opinion regarding the prognostic property of KI67 derives mostly from reviews and meta-analyses, which support its prognostic role in breast cancer [4–7, 20]. The meta-analyses by de Azambuja et al. [6] involving 12,155 patients and by Stuart-Harris et al. [7] which included over 15,000 patients represent two comprehensive analyses on this subject. These are limited, however, by reported evidence of publication bias, by significant between-study heterogeneity and by the fact that most of the included studies utilised different methodological approaches for KI67 evaluation. Furthermore, while the analysis by de Azambuja et al. [6] was limited by its inclusion of only univariate hazard ratios, that by Harris et al. [7] was limited by the small intersection between the sets of covariates in the included studies. In a population-based cohort of a cancer registry, Inwald et al. [21] examined the prognostic role of KI67 in 3658 patients for whom KI67 was routinely measured in clinical practice and reported significant associations between KI67 and overall survival [21]. An important strength of this analysis was that it utilised routinely assessed KI67 measurements in a clinical setting. But this was also limited by the heterogeneity of the KI67 analytical processes in the different laboratories involved in the study. Nonetheless, KI67 has found use in a variety of clinical and epidemiological scenarios, including its endorsement by a number of international guidelines for use in treatment decision-making in ER-positive breast cancer [1, 2] and its incorporation as part of emerging prognostic tools such as the IHC4 score [22, 23] and PREDICT, a breast cancer treatment benefit tool [24].

In this study, we evaluate the value and robustness of automated scoring of KI67 for large-scale, multicentre studies of breast cancer prognostication. We centrally generated an automated KI67 score from stained tissue microarrays (TMAs), and assessed its prognostic value overall for different subtypes of breast cancer. We also compared the prognostic performance of automated and visually derived KI67 scores in a subset of patients.

## Methods

### Study population and study design

**Fig. 1:**
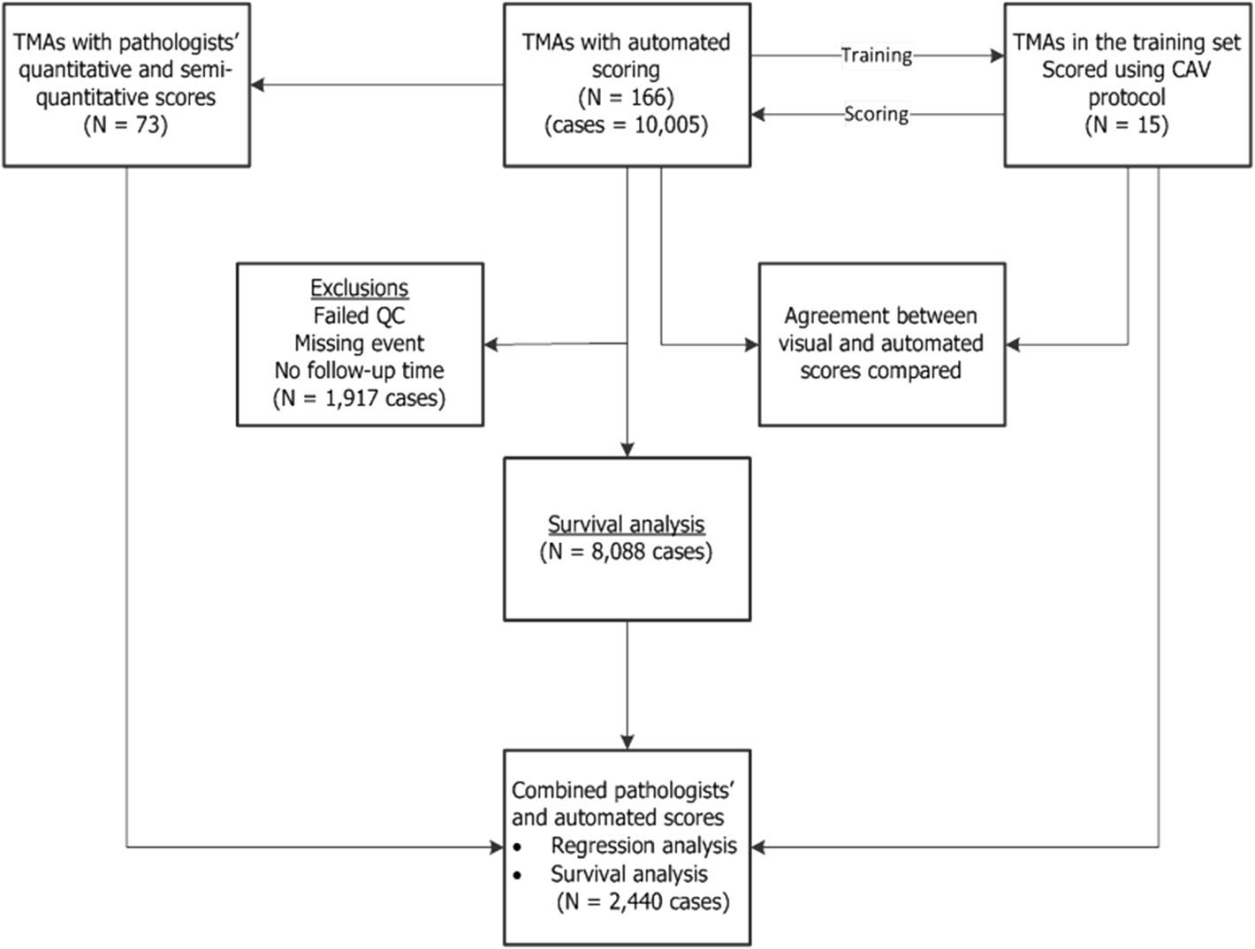
Study population and study design. We collected 166 TMAs containing 19,039 cores from 10,005 patients. Of these, 15 TMAs containing 1346 cores were selected as the training set and these were used to develop an automated scoring protocol that was validated against corresponding computer-assisted visual (*CAV*) scores. Ultimately, this protocol was applied to the scoring of all 166 TMAs. Following automated scoring, all cores that failed our priori defined quality control checks (including total nuclei count >50 and <15,000, and KI67 score = 100 %) were excluded (*N* = 946 patients). For the purpose of survival analyses, all subjects with missing follow-up/survival data were also excluded (*N* = 971 patients). As a result, a total of 8088 patients were used in the survival analysis involving automated KI67 score. Furthermore, based on a subset of patients (*N* = 2440) with pathologists’ KI67 scores in addition to the automated KI67 scores, we extrapolated a visual from an automated cut-off point and used this to compare the prognostic performance of visual and automated KI67 scores in breast cancer. *QC* quality control, *TMA* tissue microarray

This analysis was conducted within the Breast Cancer Association Consortium [25], which is a large, ongoing collaborative project involving study groups across the globe. Figure 1 shows that we collected a total of 166 TMAs containing 19,039 cores, representing 10,005 patients from 13 study groups (Additional file 1: Table S1). Ten study groups provided unstained TMAs which were then stained and digitised in the Breakthrough Core Pathology laboratory at the Institute of Cancer Research (ICR) and the academic biochemistry laboratory of the Royal Marsden Hospital (RMH), London, UK. Two groups (MARIE and PBCS) provided pre-stained TMAs which were also digitised in our centre. One study (SEARCH) provided TMA images acquired using a similar Ariol technology (a digital image acquisition and analysis system) to the one adopted for this analysis. Of the 10,005 patients, 1917 were excluded on account of failing predefined quality control checks (*N* = 946) or due to absent data on follow-up times and/or vital status (*N* = 971). As a result, a total of 8088 patients from 10 study groups with a median follow-up of 7.5 years and a total of 1401 breast cancer specific deaths were used in the survival analysis involving automated KI67. Of these, 2440 patients with pathologists’ visual KI67 scores in addition to automated KI67 scores were used to extrapolate a visual from an automated cut-off point, following which comparative survival analyses involving visual and automated KI67 scores were conducted. Information on other clinico-pathological characteristics of tumours including histological grade, nodal status, tumour size, stage, adjuvant systemic therapy (endocrine therapy and/or chemotherapy) and other IHC markers (i.e. oestrogen receptor (ER), progesterone receptor (PR) and human epidermal growth factor receptor 2 (HER2)) were obtained from clinical records. Additional Ariol HER2 data were obtained for a subset of patients with missing clinical HER2 data but for whom data on ER and PR were available (*N* = 403). All patients provided written informed consent and all participating studies gained approval from the local ethical committees and institutional review boards.

### KI67 immunostaining

Sections were dewaxed using xylene and rehydrated through graded alcohol (100, 90 and 70 %) to water. Slides were then placed in a preheated (5 min, 800 W microwave) solution of Dako Target Retrieval solution pH 6.0 (S1699), microwaved on high power for 10 min and then allowed to cool in this solution at room temperature for 10 min. In the next stage, the slides were placed on a Dako autostainer and stained using a standard protocol with Dako MIB-1 diluted 1/50 and visualised using the Dako REAL kit (K5001). The MIB-1 antibody was also adopted for the staining of the TMAs that were not part of those centrally stained at the ICR, but at varying concentrations (PBCS = 1:500; MARIE = 1:400 and SEARCH = 1:200) (Additional file 1: Table S2).

### KI67 scoring

KI67 scoring has been described previously [26], but briefly all TMAs were digitised using the Ariol 50s digital scanner. Fifteen TMAs were selected as a training set. These were scored visually by a pathologist (MA) using a computer-assisted visual (CAV) counting method and used to validate the automated method. The CAV method relied upon built-in features of the Ariol digital system to count negative and positive nuclear populations within 250 μm × 250 μm squares separated by grids. The standard CAV approach entailed the counting of at least 1000 cells across the entire spectrum of each core. In the majority of cores, more than 1000 cells were counted even though fewer than this number was counted in a small minority. Overall, cores with more than 500 cells were considered to be of satisfactory quality. The CAV method is precise, prevents double counting and was observed to have excellent intra-observer reproducibility when a random subset of cores (*N* = 111) were re-scored at an interval of 3 months from the first time they were scored (observed agreement/kappa = 96 %/0.90); good core-level agreements with two other independent scorers (observed agreement/kappa: CAV vs scorer 2 = 87 %/0.66; CAV vs scorer 3 = 84 %/0.59; scorer 2 vs scorer 3 = 89 %/0.69) were also observed in a randomly selected subset of 202 cores. Visual scoring in the external TMAs involved both quantitative and semi-quantitative methods. Each core from each patient was scored by two independent pathologists and the KI67 score for each patient was then taken as the average score from the two scorers across all cores for that patient.

The automated scoring was performed using the Ariol machine which has functionality that allows for the automatic detection of malignant and non-malignant nuclei using shape and size characteristics. Using colour deconvolution, it also distinguishes between DAB-positive and DAB-negative (haematoxylin) malignant cells. To determine the negative and positive populations of cells, an appropriate region of interest of the malignant cell population in a core was demarcated and two colours were selected to indicate positive and negative nuclear populations. The appropriate colour pixels were then selected to represent the full range of hue, saturation and intensity that was considered representative of the positive and negative nuclear classes [26]. Subsequently, the best shape parameters that discriminated malignant and non-malignant cells according to their spot width, width, roundness, compactness and axis ratio were also selected. The data were divided into a training and a validation subset and the automated and visual scoring for KI67 showed good agreement (observed agreement = 87 %; Kappa = 0.64) and discriminatory accuracy (AUC = 85 %) in the validation subset, hence allowing for the adoption of this method for the scoring of all 166 TMAs.

### Statistical methods

For patients with multiple cores from the same tumour, we used the average KI67 score across valid cores to represent the % positive cells in that tumour. Descriptive analyses of the distribution of KI67 according to clinical and pathological characteristics of the patients were conducted using the non-parametric Kruskal–Wallis equality of medians test for continuous measures and the paired chi-squared test for categorical measures. The relative survival probabilities for patients in different quartiles of the KI67 distribution were compared using Kaplan–Meier survival curves for the 10-year breast cancer specific survival (BCSS). To allow for prevalent cancers, time at risk was left-censored for study entry. It was decided, a priori, not to make any assumptions on a prognostic cut-off point for automated KI67 scores in our dataset but instead to leverage on the continuous values to observe a prognostic threshold. As a result, we performed quartile analysis by dividing the continuous KI67 scores into quartiles (Q1–Q4) and examining the prognostic differences among the different quartiles for all patients in the study. The 10-year BCSS was determined using Kaplan–Meier survival curves and Cox-proportional hazards regression models stratified by ER status (positive vs negative) and according to nodal status (positive vs negative) and other IHC markers. The univariate Cox models were partially adjusted for study group and age at diagnosis while the multivariate models had further adjustments for other known prognostic factors including histological grade, tumour size, nodal status, morphology, ER, PR, HER2 and adjuvant systemic therapy (endocrine and/or chemotherapy). In the multivariate models, missing values for other covariates were addressed using the multiple imputation plus outcome (MI+) approach [27]. Because of observed violation of the proportionality assumption of the Cox model by automated KI67, it was modelled as a time-varying covariate using an extension of the Cox model that allows for the inclusion of a coefficient (*T*) that varied as an exponential function of time. The log of the coefficient is indicative of both the direction and the magnitude of change in hazard ratio with time, such that if log *T* < 1 then hazard falls with time, while if log *T* > 1 then hazard increases with time. Known violation of the proportional hazards assumption by ER was addressed in the same way. Consistency of hazard ratio (HR) estimates across the different study groups was evaluated using the *I* ^2^ statistic, derived by performing a fixed-effect meta-analysis of study-specific HR estimates. To enable direct comparison between the visual and automated KI67 scores, we extrapolated a visual from an automated cut-off point in a linear regression model and used the resulting cut-off point for all further analyses. All analyses were conducted using STATA statistical software version 10 (StataCorp, College Station, TX, USA). Statistical tests were two-sided and *P* < 0.05 was considered statistically significant.

## Results

### Description of study population and association between automated KI67 score and other clinico-pathological characteristics of breast cancer patients (*N* = 8088)

**Fig. 2:**
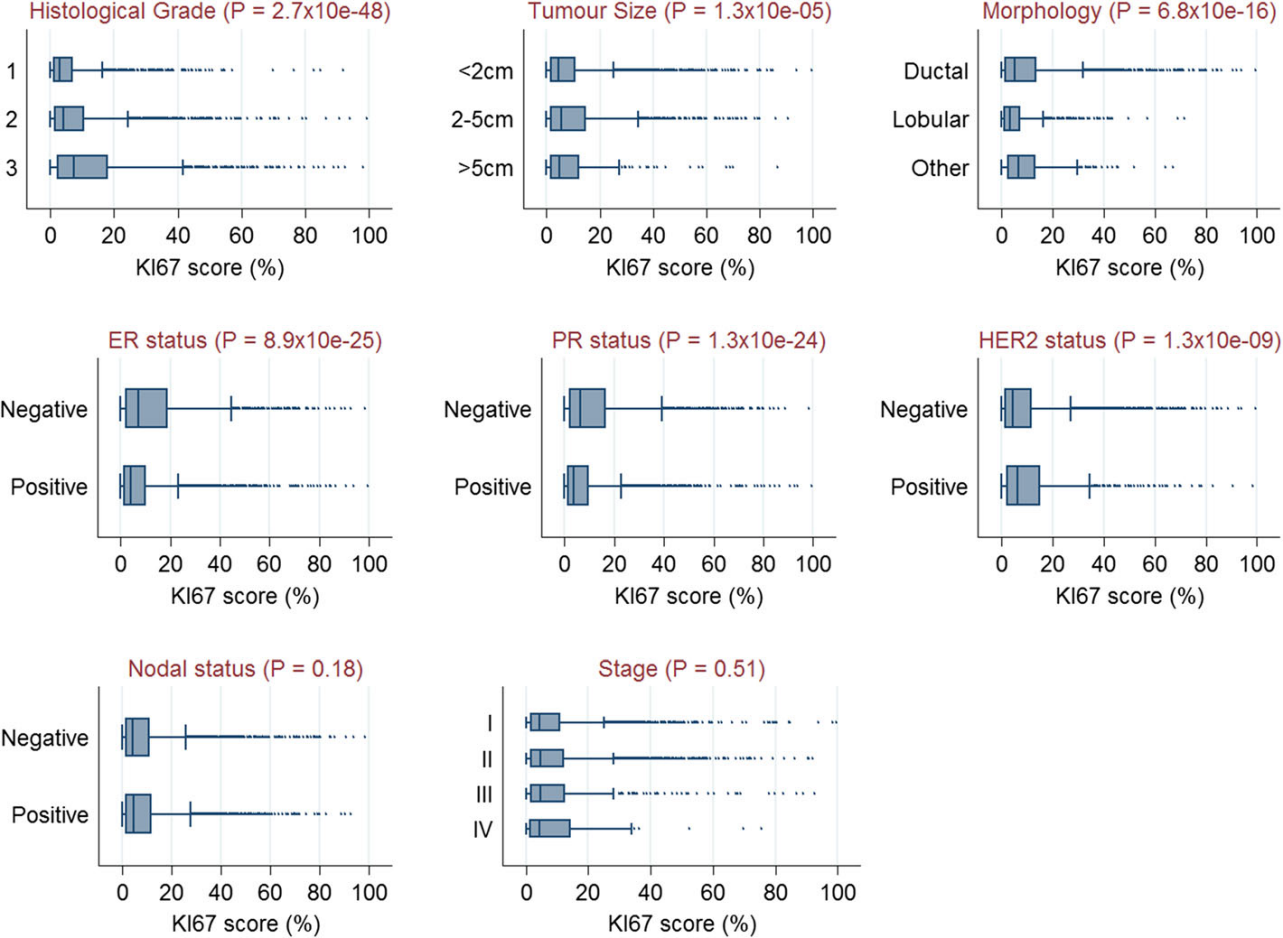
Distribution of continuous KI67 scores according to categories of other clinical and pathological variables. Significant differences were seen in the distribution of automated KI67 scores according to categories of histological grade, tumour size, morphology, ER status, PR status and HER2 status, but not nodal status or stage. *ER* oestrogen receptor, *HER2* human epidermal growth factor receptor 2, *PR* progesterone receptor

In all, a total of 143 TMAs containing 15,313 cores from 8088 patients were used in this analysis, as shown in Fig. 1. The studies included in this analysis used different TMA designs (Table 1). More than half (4431/55 %) of the patients had KI67 scores on at least two cores and evaluation of dichotomous categories revealed concordant KI67 status in 83.7 % of the patients. When we examined the distribution of continuous KI67 scores among categories of the different clinical and pathological characteristics we observed this to differ according to histological grade, tumour size, morphology, ER status, PR status and HER2 status, but not nodal status or stage at diagnosis (Fig. 2). The distribution of these characteristics for patients with high KI67 (Q4 or >12 % positive cells) and low KI67 (Q1–Q3) are shown in Additional file 1: Tables S3 and S4 for ER-positive and ER-negative patients, respectively.

**Table 1:** Description of study populations, TMA designs and patient characteristics for the 8088 patients included in this analysis

Study acronym	Country	Cases (*N*)	Age at diagnosis, mean (range)	TMAs	Cores/patient (range)	Cores/patient (average)	Cores per TMA	Core size (mm)	Total cores per study
ABCS	The Netherlands	885	43 (19–50)	24	1–6	2.77	15–328	0.6	2449
ESTHER	Germany	252	62 (50–75)	6	1–2	1.83	78–91	0.6	461
KBCP	Finland	266	59 (30–92)	12	1–3	2.72	63–94	1.0	724
MARIE	Germany	808	62 (50–75)	27	1–5	1.84	32–92	0.6	1490
MCBCS	USA	437	58 (22–87)	7	1–8	3.73	131–301	0.6	1630
ORIGO	The Netherlands	348	53 (22–87)	9	1–9	2.85	67–223	0.6	991
PBCS	Poland	1068	56 (27–75)	22	1–2	2.21	66–145	1.0	2358
RBCS	The Netherlands	225	45 (25–84)	6	1–5	2.85	134–199	0.6	642
SEARCH	United Kingdom	3491	52 (24–70)	24	1–3	1.16	120–167	0.6	4037
kConFab	Australia/New Zealand	308	45 (20–77)	6	1–2	1.72	65–114	0.6	531
Total		8088	53 (19–92)	143	1–9	1.89	15–328	0.6–1.0	15,313

*TMA* tissue microarray

### Association between automated KI67 score and 10-year BCSS among 8088 patients

**Fig. 3:**
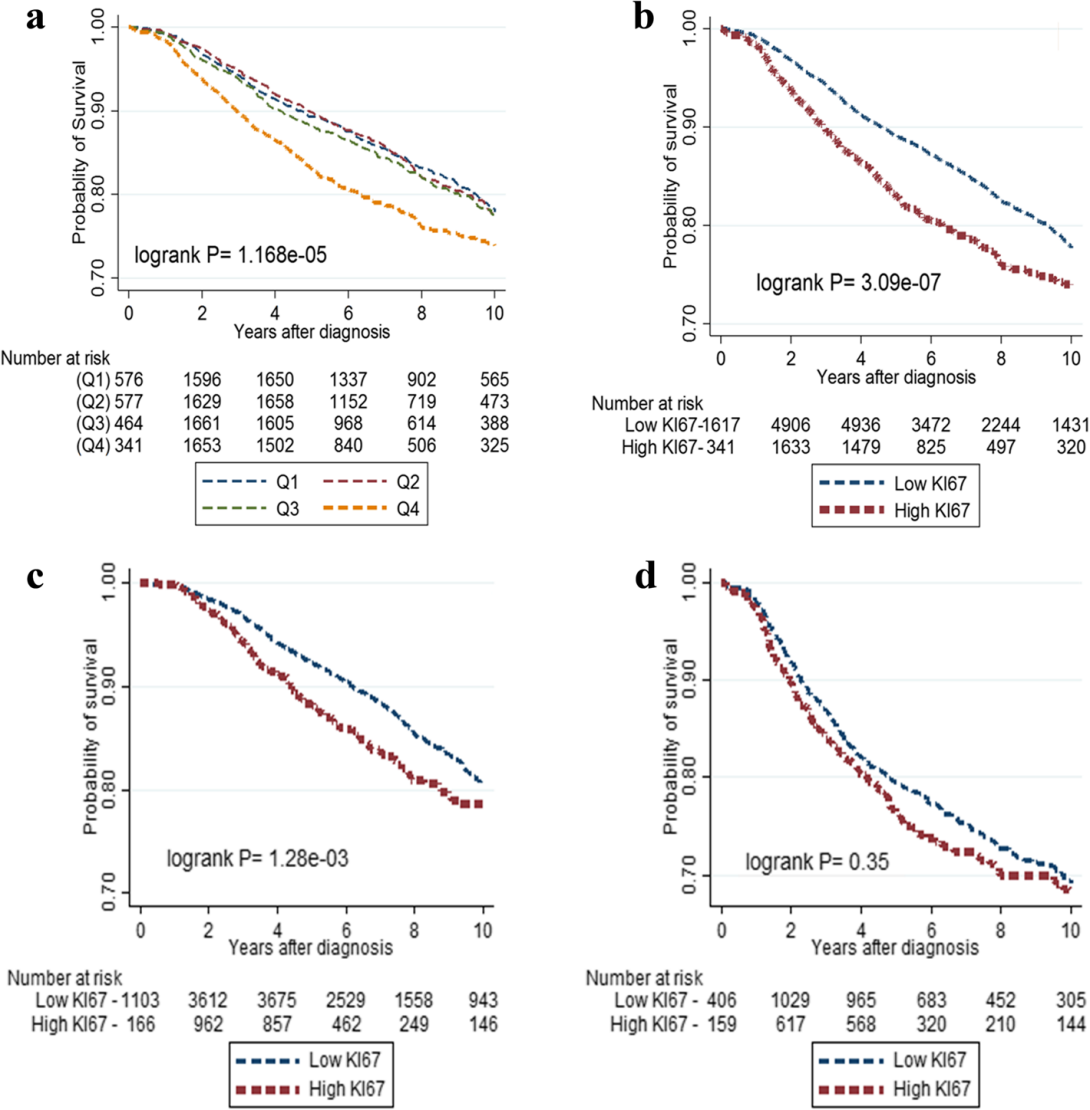
Kaplan–Meier survival curves for the 10-year BCSS according to strata of automated KI67 scores, overall and by ER status. KM survival curves for the association between KI67 and 10-year BCSS among: (**a**) quartiles of KI67 (Q1, <25th percentile; Q2, 25th–50th percentile; Q3, >50th to 75th percentile; and Q4, >75th percentile; *N* = 8088); (**b**) dichotomous categories of KI67 (≤12 %/low and >12 %/high) overall (*N* = 8088 patients); (**c**) ER-positive cancers (*N* = 5520 patients); and (**d**) ER-negative cancers (*N* = 2049 patients)

Using continuous measures of KI67 categorised into quartiles, we observed poorest survival in the highest quartile, corresponding to 12 % positive cells, but little difference in survival between the other three (Q1–Q3) quartiles (log-rank *P* = 1.2 × 10^−5^; Fig. 3a). As a result, the continuous KI67 value was dichotomised at the threshold of 12 % in subsequent analyses. High KI67 was significantly associated with worse 10-year BCSS overall (log-rank *P* = 3.1 × 10^−7^) among ER-positive cancers (log-rank *P* = 1.3 × 10^−3^) but not ER-negative cancers (log-rank *P* = 0.35) (Fig. 3b–d, respectively). Similarly, in multivariate models, high KI67 expression was significantly associated with worse 10-year BCSS among ER-positive cancers (HR at baseline = 1.96; 95 % CI = 1.31–2.93) but not ER-negative breast cancers (HR = 1.23; 95 % CI = 0.86–1.77; *P*-heterogeneity = 0.064) (Table 2). Further stratification of ER-positive cancers according to nodal status showed that high KI67 was associated with worse survival in both node-negative and node-positive cancers in multivariate analysis (node-negative 2.47 (1.16–5.27); node-positive 1.74 (1.05–2.86); *P*-heterogeneity = 0.67) (Table 2). The association between KI67 and survival was significant among ER-positive patients who did not receive chemotherapy (1.95 (1.18–3.21); *P* = 0.009) but not among those who did (1.89 (0.84–4.29); *P* = 0.124; *P*-heterogeneity = 0.60). We found no evidence of between-study heterogeneity in estimates of HR for ER-positive patients (*I* ^2^ = 0.0 %, *P* = 0.94) or ER-negative patients (*I* ^2^ = 0.0 %, *P* = 0.86) (Additional file 2: Figure S1). Among hormone receptor-positive breast cancers, the HR for KI67 was not significantly different according to HER2 status (Table 2; *P*-heterogeneity = 0.270). Modest evidence for a poorer prognosis among high, relative to low, KI67 was also seen for triple-negative breast cancers (1.70 (1.02–2.84); *P* = 0.04). No significant associations with prognosis were found for KI67 among HER2-positive (i.e. ER^–^/PR^–^/HER2^+^) breast cancers (0.91 (0.60–1.36)) (Table 2).

**Table 2:** Hazard ratio (HR) and 95 % CI for the association between automated KI67 score and 10-year BCSS in partially and fully adjusted models: analysis stratified overall and according to ER, nodal status and other immunohistochemical markers (*N* = 8088 patients)

			Partially adjusted^a^		Fully adjusted^b^	
	Patients (*N*)	Deaths (*N*)	HR (95 % CI)	*P* value	HR (95 % CI)	*P* value
All cancers						
Low KI67	6093	1030	1.00 (referent)		1.00 (referent)	
High KI67	1995	371	2.32 (1.79–3.00)	6.34 × 10^−11^	1.47 (1.13–1.92)	0.004
*T* ^c^			0.89 (0.84–0.94)	3.20 × 10^−5^	0.93 (0.87–0.98)	0.010
ER-positive						
Low KI67	4379	615	1.00 (referent)		1.00 (referent)	
High KI67	1141	166	2.47 (1.63–3.72)	8.45 × 10^−6^	1.96 (1.31–2.93)	0.001
*T*			0.88 (0.81–0.96)	0.003	0.89 (0.82–0.97)	0.006
ER-negative						
Low KI67	1271	320	1.00 (referent)		1.00 (referent)	
High KI67	778	188	1.38 (0.97–1.97)	0.072	1.23 (0.86–1.77)	0.248
*T*			0.94 (0.86–1.02)	0.155	0.94 (0.87–1.03)	0.199
ER-positive/node-positive						
Low KI67	1550	350	1.00 (referent)		1.00 (referent)	
High KI67	408	94	2.22 (1.31–3.77)	0.003	1.74 (1.05–2.86)	0.031
*T*			0.91 (0.82–0.99)	0.044	0.91 (0.82–1.01)	0.075
ER-positive/node-negative						
Low KI67	2399	205	1.00 (referent)		1.00 (referent)	
High KI67	561	55	3.17 (1.43–6.99)	0.004	2.47 (1.16–5.27)	0.019
*T*			0.84 (0.71–0.98)	0.034	0.86 (0.73–0.99)	0.048
HRP/HER2^–^						
Low KI67	3332	462	1.00 (referent)		1.00 (referent)	
High KI67	831	114	1.69 (1.26–2.27)	2.42 × 10^−4^	1.49 (1.10–2.00)	0.009
*T*			0.94 (0.90–0.98)	0.004	0.94 (0.90–0.98)	0.004
HRP/HER2^+^						
Low KI67	421	82	1.00 (referent)		1.00 (referent)	
High KI67	157	36	1.96 (1.28–3.00)	9.70 × 10^−4^	1.59 (1.03–2.45)	0.035
*T*			0.94 (0.90–0.98)	0.004	0.94 (0.90–0.98)	0.004
HRN/HER2^–^ (triple-negative)						
Low kI67	565	142	1.00 (referent)		1.00 (referent)	
High KI67	436	107	1.75 (1.06–2.90)	0.028	1.70 (1.02–2.84)	0.044
*T*			0.86 (0.75–0.98)	0.031	0.86 (0.75–0.99)	0.031
HRN/HER2^+^ (HER2-enriched)						
Low KI67	227	85	1.00 (referent)		1.00 (referent)	
High KI67	149	48	0.76 (0.37–1.55)	0.450	0.75 (0.36–1.57)	0.455
*T*			1.08 (0.90–1.29)	0.396	1.07 (0.90–1.29)	0.435

^a^Partially adjusted models—adjusted for age at diagnosis and study group

^b^Fully adjusted models—further adjustment for histological grade, tumour size, nodal status, morphology, PR, HER2, systemic therapy (endocrine and/or chemotherapy) and (for the model involving all patients) ER status. This model was based on 20 imputations performed to address missing values on other covariates

^c^Log of time-varying coefficient (if *T* < 1 then hazard falls with time, and if *T* >1 then hazard increases with time)

*HRP* hormone receptor-positive, *HRN* hormone receptor-negative, *ER* oestrogen receptor, *HER2* human epidermal growth factor receptor 2, *PR* progesterone receptor

### Comparison of 10-year BCSS among 2440 patients with both visual and automated quantitative KI67 scores

The automated cut-off point of 12 % positive cells corresponded to a visual cut-off point of 24.2 % based on a linear regression model comprising patients with quantitative data on both methods. The visual cut-off was rounded up to a cut-off point of 25 %. Strong evidence (*P* < 0.0001) in support of a positive linear correlation (*r* = 0.63) between automated and visual scores was observed and continuous automated scores showed good discriminatory accuracy against the visually determined binary classes (AUC = 82 %, 95 % CI = 80–84 %)(Additional file 3: Figure S2)﻿. Twenty-six percent of the patients were classified as having high visual KI67, in contrast to 29 % for the automated KI67 scores; cross-classification of visual and automated categories revealed better specificity (84 %) than sensitivity (65.6 %) for the automated score in classifying visually determined categories (Additional file 1: Table S5). High KI67 was associated with worse survival in Kaplan–Meier curves based on both automated (log-rank *P* = 9.8 × 10^−6^) and visual (log-rank *P* = 3.8 × 10^−14^) KI67 scores even though attenuation of the difference between strata was observed for automated KI67 scores (Additional file 4: Figure S3). In two separate models for visual and automated KI67 scores each adjusted for age at diagnosis and study group we observed stronger evidence for an association between KI67 and survival for the visual KI67 score than for the automated KI67 score (Table 3). Analysis of model fit revealed similar parameters for both scores, however, especially in ER-positive breast cancers (AIC/BIC: visual = 2656/2618; automated = 2675/2638) (Table 3). When we performed further adjustments for other prognostic factors in multivariate Cox models of imputed datasets, we observed both visual and automated KI67 scores to be significantly associated with survival for all patients (HR (95 % CI): visual = 1.75 (1.23–2.49); automated = 1.61 (1.14–2.28)) and for ER-positive patients (visual = 2.30 (1.34–3.94); automated = 2.10 (1.28–3.47)), but not for ER-negative patients (visual = 1.63 (0.97–2.72); automated = 1.28 (0.79–2.05)) (Table 3).

**Table 3:** Univariate (partially adjusted) and multivariate (fully adjusted) hazard ratio (HR) and 95 % CI for the associations between automated and visual KI67 scores with survival in breast cancer (*N* = 2440)

	Partially adjusted model^a^								Fully adjusted model^b^			
	Visual				Automated				Visual		Automated	
	Patients (*N*)	Deaths (*N*)	HR (95 % CI)	*P* value	Patients (*N*)	Deaths (*N*)	HR (95 % CI)	*P* value	HR (95 % CI)	*P* value	HR (95 % CI)	*P* value
Overall												
Low KI67	1804	116	1.00 (referent)		1728	125	1.00 (referent)		1.00 (referent)		1.00 (referent)	
High KI67	636	78	2.40 (1.92–3.01)	2.20 × 10^−14^	712	69	1.67 (1.33–2.10)	1.30 × 10^−5^	1.75 (1.23–2.49)	0.002	1.61 (1.14–2.28)	0.007
AIC^c^			5050.4				5087.2					
BIC^c^			5090.8				5127.6					
ER-positive												
Low KI67	1,337	69	1.00 (referent)		1241	69	1.00 (referent)		1.00 (referent)		1.00 (referent)	
High KI67	282	27	2.40 (1.72–3.33)	2.00 × 10^−7^	378	27	1.47 (1.05–2.04)	0.024	2.30 (1.34–3.94)	0.002	2.10 (1.28–3.47)	0.004
AIC			2618.8				2638.2					
BIC			2656.8				2675.8					
ER-negative												
Low KI67	357	39	1.00 (referent)		392	48	1.00 (referent)		1.00 (referent)		1.00 (referent)	
High KI67	331	48	1.84 (1.30–2.62)	6.10 × 10^−4^	296	39	1.44 (1.02–2.04)	0.043	1.62 (0.97–2.72)	0.066	1.28 (0.79–2.05)	0.312
AIC			1755.7				1763.4					
BIC			1787.4				1795.1					

^a^Partially adjusted model—adjusted for age at diagnosis and study group only

^b^Fully adjusted model—further adjustment for other prognostic factors including histological grade, tumour size, nodal status, ER, PR, HER2, morphological subtype and systemic therapy (endocrine and/or chemotherapy). This model was based on 20 imputations performed to address missing values on other covariates

^c^Model fit parameter

*AIC* Akaike information criterion, *BIC* Bayesian information criterion, *ER* oestrogen receptor, *HR* hazard ratio, *HER2* human epidermal growth factor receptor 2, *PR* progesterone receptor

## Discussion

Findings from our analysis provide strong evidence in support of a prognostic relationship for automated KI67 scoring in ER-positive (node-negative and node-positive) patients that is independent of tumour grade and other prognostic factors. Even though our data suggested a larger magnitude of the association between KI67 and survival among the node-negative patients, the difference between node-positive and node-negative was not statistically significant. Involving over 8000 patients from multiple centres internationally, this represents the largest study that has evaluated the prognostic value of automated KI67 scoring in breast cancer to date. Furthermore, the large sample size allowed us to evaluate its prognostic value in a number of breast cancer subtypes including ER^+^ (node-negative and node-positive), ER^–^, ER^+^ and/or PR^+^ (HER2^+^ or HER2^–^), ER^–^/PR^–^ and HER2^+^ (i.e. HER2-enriched) and triple-negative breast cancers.

Our findings suggest that automated KI67 scoring is an analytically valid approach to generating KI67 scores. This is particularly noteworthy given the growing need to incorporate measures of KI67 in prognostic tools such as the IHC4 score and PREDICT [23, 24]. These tools are relatively cheap, readily available and utilise routinely measured IHC markers and, in the case of PREDICT, other routinely available patient data to provide information that can help clinicians and patients make informed decisions regarding the course of treatment. It is acknowledged that prognostication in breast cancer is becoming increasingly more sophisticated and that a number of multigene assays [28, 29] have been validated for this purpose; however, their costs and proprietary concerns limit their use in a large number of settings. Moreover, findings from previous studies suggest that some multigene assays may not perform better than routinely measured IHC markers. For instance, Cuzick et al. [23] reported similar prognostic properties for the Genomic Health recurrence score (GHI-RS, Oncotype DX), a 21-gene panel test, and the IHC4 score in their analysis of 1125 women from the TransATAC study, and notably KI67 was assessed by image analysis in that study [23]. Nonetheless, the relative performance of visual and automated KI67 scores in relation to the IHC4 score or PREDICT can only be assessed in studies that are specifically designed for that purpose.

In addition to lack of analytical validity, the prognostic performance of KI67 has also been questioned due to the design and analysis of studies that have reported previously on this protein [3]. Our evaluation is a large-scale, multicentre analysis which has adopted the recommended laboratory processes for the staining and scoring of KI67 [8]. All TMAs in our analysis were stained using the MIB1 antibody (even though not all of them were centrally stained in our centre) and scored using a single automated algorithm. Our estimates of ~2-fold and ~1.5-fold increased risk of mortality at baseline for high versus low KI67 in univariate and multivariate analyses, respectively, are similar to those reported by de Azambuja et al. (HR = 1.95) and Harris et al. (HR = 1.42) [6, 7] in their univariate and multivariate meta-analyses, respectively. Stratification of our analysis according to other IHC markers (in addition to ER) showed automated KI67 to be prognostic in hormone receptor-positive cancers. These findings, together with our observation of the prognostic value of KI67 in both node-negative and node-positive ER-positive patients, support the decision by the St Gallen International Expert Consensus to endorse KI67 for treatment decision-making in ER-positive early (1–3 axillary nodes) breast cancer patients [1]. We also observed modest evidence in support of poorer survival outcomes among high, relative to low, KI67 expressing triple-negative subtypes of breast cancer. This finding is in support of a previous report by Keam et al. [30]. Our population of triple-negative breast cancers (*N* = 1001), however, was 9.5 times larger than that of Keam et al. (*N* = 105).

Comparative analysis of visual and automated KI67 scores showed a stronger survival association for the visual over the automated scores; however, differences were generally modest. Given the advantages of automated versus visual scoring in terms of its potential for standardisation, reproducibility and throughput, automated methods appear to be promising alternatives to visual scoring for KI67 assessment. A potential limitation to the adoption of automated KI67 scoring in the clinical setting is that misclassification of positive nuclei as negative or malignant nuclei as benign could lead to attenuation of prognostic associations, an observation that has been reported previously for ER and PR [31] and one which we have also observed for KI67 in this analysis. This can be mitigated, however, by stringent quality control processes or by the adoption of a synergistic approach that combines the benefits of both the automated and visual scoring methods. One such approach is the CAV scoring method which we developed for the visual counting of negative and positive malignant nuclei. This approach, a variation of which has been reported previously [15], exploits the advantages of both visual and digital imaging tools by enabling the visual counting of KI67-positive cells in well-defined areas of a tumour within a computer microenvironment. This method is limited, however, by the observation that it is time consuming; as such, it may not be efficient if adopted for the large-scale scoring of KI67 in epidemiological studies, clinical trials or biomarker discovery studies. Nonetheless, efforts are currently underway to standardise the methods for the visual scoring of KI67 in core-cuts.

We centrally generated KI67 scores on TMAs and determined a threshold of 12 % positive cells of prognostic relevance in our study population. However, due to possible variations in the distribution of KI67 scores according to specimen type and among different laboratories, this cut-off point may not apply to other types of clinical samples or to other laboratories. As a result, pending international standardisation of the KI67 analytical processes, setting local laboratory-specific cut-off points as recommended by international guidelines [1] remains a pragmatic approach to determining ‘high’ and ‘low’ KI67. Furthermore, although our automated cut-off point of 12 % positive cells was determined to correspond to a visual score of 25 %, this may be related, at least in part, to the fact that automated systems generally count more cells than the visual evaluator, a reason that has been proposed to explain differences in KI67 scores between visual and automated scoring and different automated scoring approaches [26]. Nonetheless, findings from a recent meta-analysis that assessed the prognostic value of different cut-off levels of KI67 suggest that a visual cut-off point >25 % provides greater discrimination in mortality risk than other cut-off points [32].

Some limitations of our analysis include the lack of data on specific chemotherapeutic or endocrine agents received by each patient, as a result of which we were unable to account for the impact of a specific treatment regimen on survival or to examine whether or not KI67 is predictive of response to specific chemotherapeutic and/or endocrine agents. We were, however, able to account for whether or not patients received adjuvant systemic treatment in all our analyses because more than two-thirds of the patients had information on treatment. This also allowed us to perform stratified analysis according to whether or not chemotherapy was administered. Also, we did not have data on disease-free survival which may have been a more informative end point than BCSS in early breast cancer. Our assessment of KI67 on TMAs may mean that direct inference cannot be drawn from our findings on other types of clinical samples, especially whole sections [8]. This is because KI67 scores are speculated to be lower for TMAs than for whole sections and not many studies have assessed the correlation between KI67 scores on TMAs and those on whole sections. However, one such study by Kobierzycki et al. [33] involving 51 archival paraffin blocks of invasive ductal carcinoma showed excellent correlation (*r* = 0.91) between the TMAs and whole sections. Their paper utilised three 0.6 mm core punches, however, and this may explain the high correlation between KI67 scores on TMAs and whole sections that was observed in that study. Nonetheless, the fact that more than half (4431/55 %) of the patients in our analysis had KI67 scores on two or more cores, with 83 % of these showing concordant KI67 status, should limit the impact of intra-tumour heterogeneity of KI67 scores on our findings.

## Conclusion

Our large, multicentre study indicates that automated KI67 scoring provides prognostic information in breast cancer that is independent of standard parameters. In view of its potential for standardisation, throughput and reproducibility, the automated method appears to be a promising alternative to visual scoring for KI67. These findings are important given the increasing need to incorporate measures of KI67 as part of tools that are needed to refine prognostic scores for breast cancer patients; this is especially relevant for patients with ER-positive, node-negative tumours, in order to aid decisions on providing adjuvant chemotherapy. However, further work is needed to standardise the staining and scoring protocols for KI67. In doing so, the potential benefits and drawbacks of automated versus visual scoring systems should merit consideration. In light of this we welcome ongoing efforts by the International Working Party on KI67 in Breast Cancer aimed at standardisation of the analytical processes for KI67.

## Additional files

Additional file 1: Is Table S1 presenting a description of study populations, Table S2 presenting KI67 immunohistochemistry reagents and antigen retrieval protocols according to study groups, Table S3 presenting the association of clinical and pathological characteristics with high (>12 %) and low (≤12 %) KI67 categories among 5520 ER-positive breast cancer cases, Table S4 presenting the association of clinical and pathological characteristics with high (>12 %) and low (≤12 %) KI67 categories among 2049 ER-negative breast cancer cases, Table S5 presenting cross-classification of visual and automated KI67 score categories, Table S6 presenting a multivariate model for the association of KI67 with 10-year BCSS among 5520 ER-positive patients, and Table S7 presenting a multivariate model for the association of KI67 with 10-year BCSS among 2049 ER-negative patients. (PDF 237 kb)
Additional file 2: Is Figure S1 showing meta-analysis of study-specific HR stratified by ER status. (TIF 1182 kb)
Additional file 3: Is Figure S2 showing ROC curve for the discriminatory accuracy of continuous automated KI67 scores against binary visual categories (≤25 % and > 25 %) based on a subset of patients with data on both visual and automated KI67 scores. (TIF 25 kb)
Additional file 4: Is Figure S3 showing Kaplan–Meier survival curves for the 10-year BCSS according to strata (high and low) of automated and visual KI67 scores (*N* = 2440). (TIF 1271 kb)

## Abbreviations

AUC: Area under the curve
BCAC: Breast Cancer Association Consortium
BCSS: Breast cancer specific survival
CAV: Computer-assisted visual
DAB: Diaminobenzidine
ER: Oestrogen receptor
GHI-RS: Genomic Health recurrence score
HER2: Human epidermal growth factor receptor 2
HR: Hazard ratio
ICC: Intra-class correlation
ICR: Institute of Cancer Research
IHC: Immunohistochemical
MI: Multiple imputation
PR: Progesterone receptor
ROC: Receiver operating characteristic
TMA: Tissue microarray
