# Supplementary material for: Prognostic value of automated KI67 scoring in breast cancer: a centralised evaluation of 8088 patients from 10 study groups
Source: Breast Cancer Res. 2016 Oct 18;18:104. doi: 10.1186/s13058-016-0765-6 (PMC5070183; doi:10.1186/s13058-016-0765-6)
Supplement: Additional file 1: — Is Table S1 presenting a description of study populations, Table S2 presenting KI67 immunohistochemistry reagents and antigen retrieval protocols according to study groups, Table S3 presenting the association of clinical and pathological characteristics with high (>12 %) and low (≤12 %) KI67 categories among 5520 ER-positive breast cancer cases, Table S4 presenting the association of clinical and pathological characteristics with high (>12 %) and low (≤12 %) KI67 categories among 2049 ER-negative breast cancer cases, Table S5 presenting cross-classification of visual and automated KI67 score categories, Table S6 presenting a multivariate model for the association of KI67 with 10-year BCSS among 5520 ER-positive patients, and Table S7 presenting a multivariate model for the association of KI67 with 10-year BCSS among 2049 ER-negative patients. (PDF 237 kb) [file 13058_2016_765_MOESM1_ESM.pdf]

Supplementary Table 1: Description of study populations

Study name	Acronym	Country	Design
Amsterdam Breast Cancer Study	ABCS	Netherlands	This is a hospital-based case-control study involving 1,084 consecutive breast cancer patients, unselected for family history and/or age who were recruited from three different centres in Netherlands including two academic centres in Leiden and Rotterdam and one general hospital in Leiden. Recruitment was done between October 1996 and July 1, 2004 subsequent to which the subjects have been followed up since then. Patients with a prior history of primary breast cancer and, in the Leiden study, those above the age of 70 years were excluded (1).
Spanish National Cancer Centre Breast Cancer Study	CNIO	Spain	This is a hospital-based case-control study involving populations from Spain. Women with a diagnosis of breast cancer were recruited between 2000 and 2004. In all, a total of 864 cases were recruited. 574 of these were recruited from three Spanish public hospitals including 263 (46%) from Monte Naranco hospital in Oviedo, Spain; 187 (33%) from the Fundación Jimenez Diaz and 124 (22%) from Hospital La Paz. The latter two hospitals are located in Madrid, Spain. Another 290 cases were recruited from the Spanish National Cancer Centre family cancer clinic for genetic testing. A total of 845 women free of breast cancer were also recruited as controls (2).
ESTHER Breast cancer study	ESTHER	Germany	ESTHER study is a mixed hospital and population-based case-control study that is focused on assessing morbidity and frailty in old age. Cases were women between the ages of 50-75 years with histologically diagnosed breast cancer in all hospitals in the state of Saarland, Germany from 2001 to 2003 that were recruited during their first stay in the hospital. Controls were women from the hospital and from the general population (3).
Kuopio Breast Cancer Project	KBCP	Finland	This is a mixed hospital- and population-based case-control study on diet and breast cancer. Cases were recruited from among women who were invited to the Kuopio University Hospital following an initial diagnosis of breast lump or suspected breast disease between April 1990 and December 1995. Of these women, all those with histologically confirmed breast cancer were recruited into the study. During the enrolment period, about 350 women with a diagnosis of breast lump or suspected breast disease were recruited into the Kuopio study annually, out of these, an annual average of 85 breast cancers were diagnosed. Controls were randomly selected from the population register covering the same geographical region as the cases (4).
Kathleen Cuningham Foundation Consortium for Familial Breast Cancer	kConFab	Australia and New Zealand	This is an Australian-based multidisciplinary and collaborative study that is aimed at collecting relevant data and biological samples from families with cases of breast and/or ovarian cancer. Starting 1997, several individuals including those affected and unaffected by breast cancer have been recruited into the study from family cancer clinics around Australia and New Zealand. All reports of cancer in a family are verified through a variety of means including the medical records, state-based cancer registries and by other systematic searches of Australian cancer registry records. Familial breast cancer patients were recruited in the clinics while controls were women from a population-based case-control study of ovarian cancer (5).
Mammary Carcinoma Risk Factor Investigation.	MARIE	Germany	Population-based case-control study of breast cancer in Northern and Southern Germany. Cases from this study were incident and prevalent cases diagnosed from 2001-2005 in the study region of Hamburg in Northern Germany and from 2002-2005 in the study region of Rhein-Neckar-Karlsruhe in Southern Germany. Controls were randomly drawn from population registries and frequency matched by birth year and study region to the case. Controls were recruited from 2002 to 2006 (6)
Mayo Clinic Breast Cancer Study	MCBCS	USA	An on-going study; cases are unselected, clinic based, series of breast cancer patients diagnosed within the previous 6 months no prior history of cancer (except non-melanoma skin cancer) who were seen in the division of Medical Oncology between February 1, 2001 and June 2005 (7)
Leiden University Medical Centre Breast Cancer Study	ORIGO	Netherlands	Cases in this study were a consecutive series of breast cancer cases unselected for family history recruited from three centres in south west Netherlands (including two academic cancer centres in Leiden and Rotterdam, and one general hospital in Leiden) between October 1, 1996 and July 1, 2002 (8).
NCI Polish Breast Cancer Study	PBCS	Poland	A population-based case-control study set in Poland over a three year period with a total of 2,386 cases and 2,502 controls. Cases were women with histologically

			confirmed breast cancer and between the ages of 20 and 74 years. Controls were randomly selected using the Polish electronic system – a directory of all Polish residents (9).
Prospective study of outcomes in sporadic versus hereditary breast cancer	POSH	UK	A population-based case-control study set in Poland over a three year period with a total of 2,386 cases and 2,502 controls. Cases were women with histologically confirmed breast cancer and between the ages of 20 and 74 years. Controls were randomly selected using the Polish electronic system – a directory of all Polish residents (10).
Rotterdam Breast Cancer Study	RBCS	Netherlands	The RBCS is a hospital based case-control study comprising 180 cases and based in the Netherlands. Cases were subjects with a histologically confirmed diagnosis of breast cancer while controls were other hospital patients who were in the hospital for causes unrelated to breast cancer (11).
Study of Epidemiology and Risk Factors in Cancer Heredity	SEARCH	UK	This is an on-going population-based study; with breast cancer ascertained through the East Anglian Cancer Registry. Cases were those diagnosed with invasive breast cancer below the age of 55 years between 1991 and mid-1996 and still alive in 1996 when the study began (prevalent cases) together with women with invasive breast cancer diagnosed at age <70 years from the mid 1996 onwards (12)
Breakthrough Generations Study	BGS	UK	The BGS is a large, ongoing, prospective cohort study in the United Kingdom which began in 2003 and has so far recruited over 110,000 women. The UKBGS is unique its design by virtue of the age range of subjects at recruitment (16-102) and generational nature of its recruitment process – where subjects who are enrolled in the process volunteer to enrol a new generation of study subjects: these can be friends and/or family members. Extensive questionnaire information was obtained from these patients including blood samples and anthropometric measurements and this process is repeated every three and half years. Cases in the BCAC study are those individuals who developed breast cancer during the follow-up period (13).

Supplementary Table 2: KI67 immunohistochemistry reagents and antigen retrieval protocols according to study groups

Site	Clone	Clonality	Source	Dilution	Antigen retrieval
ICR	MIB-1	Mouse monoclonal	DAKO	1:50	Dako Target Retrieval Solution, pH6, 20 minutes
PBCS	MIB-1	Mouse monoclonal	DAKO	1:500	Tris-EDTA buffer, pH9, 20 minutes
SEARCH	MIB-1	Mouse monoclonal	DAKO	1:200	Tris-EDTA buffer, pH9, 30 minutes
MARIE	MIB-1	Mouse monoclonal	DAKO	1:400	Tris-EDTA buffer, pH9, 10 minutes

Supplementary Table 3: Association of clinical and pathological characteristics with high (>12%) and low (≤12%) KI67 categories among 5,520 ER positive breast cancer cases

Characteristic	Low KI67 (≤12%) N = 4,379	%	High KI67 (>12%) N = 1,141	%	P
**Age at diagnosis**					
<35	125	2.86	22	1.93	
35-50	1,420	32.44	355	31.11	
>50-65	2,045	46.72	567	49.69	
>65	787	17.98	197	17.27	
Missing	2				0.147
**Histological grade**					
Low grade	1,155	29.25	189	18.42	
Intermediate grade	2,083	52.75	542	52.83	
High grade	711	18.00	295	28.75	
Missing	430		115		1.67E-18
**Stage**					
I	1,855	46.69	463	46.07	
II	1,827	45.99	465	46.27	
III	245	6.17	58	5.77	
IV	46	1.16	19	1.89	
Missing	406		136		0.091
**Morphology**					
Ductal	1,961	70.34	600	75.95	
Lobular	551	19.76	94	11.90	
Other	276	9.90	96	12.15	
Missing	1,591		351		1.49E-06
**Tumour size**					
<2cm	2,343	66.32	590	59.90	
>2cm and <5cm	1,093	30.94	366	37.16	
≥ 5cm	97	2.75	29	2.94	
Missing	846		156		8.14E-04
**Node status**					
Negative	2,399	60.75	561	57.89	
Positive	1,550	39.25	408	42.11	
Missing	430		172		0.104
**PR expression**					
Negative	762	18.58	218	21.08	
Positive	3,340	81.42	816	78.92	
Missing	277		107		0.066
**HER2 expression**					
Negative	3,033	89.18	731	83.35	
Positive	368	10.82	146	16.65	
Missing	978		264		2.22E-06
**Chemotherapy***					
Not received	2,338	78.09	505	74.70	
Received	656	21.91	171	25.30	
Missing	1,385		404		0.057
**Endocrine therapy**					
Not received	984	29.04	253	27.77	
Received	2,404	70.96	658	72.23	
Missing	991		230		0.452

*Chemotherapy is adjuvant

Supplementary Table 4: Association of clinical and pathological characteristics with high (>12%) and low (≤12%) KI67 categories among 2,049 ER negative breast cancer cases

Characteristic	Low KI67 (≤12%) N = 1,271	%	High KI67 (>12%) N = 778	%	P
**Age at diagnosis**					
<35	70	5.51	57	7.33	
35-50	514	40.44	283	36.38	
>50-65	550	43.27	334	42.93	
>65	137	10.78	104	13.37	
Missing					0.060
**Histological grade**					
Low grade	121	10.37	21	2.97	
Intermediate grade	416	35.65	160	22.63	
High grade	630	53.98	526	74.40	
Missing	104		71		2.32E-19
**Stage**					
I	386	34.01	214	31.94	
II	650	57.27	388	57.91	
III	84	7.40	59	8.81	
IV	15	1.32	9	1.34	
Missing	136		108		0.527
**Morphology**					
Ductal	665	80.80	462	86.68	
Lobular	89	10.81	20	3.75	
Other	69	8.38	51	9.57	
Missing	448		245		1.73E-05
**Tumour size**					
<2cm	531	53.96	297	46.85	
>2cm and <5cm	400	40.65	315	49.68	
≥ 5cm	53	5.39	22	3.47	
Missing	287		144		9.15E-04
**Node status**					
Negative	620	53.13	391	56.34	
Positive	547	46.87	303	43.66	
Missing	104		84		0.179
**PR expression**					
Negative	937	77.95	653	88.24	
Positive	265	22.05	87	11.76	
Missing	69		38		1.09E-08
**HER2 expression**					
Negative	710	73.20	480	75.35	
Positive	260	26.80	157	24.65	
Missing	301		141		0.335
**Chemotherapy***					
Not received	421	54.89	208	49.17	
Received	346	45.11	215	50.83	
Missing	504		355		0.058
**Endocrine therapy**					
Not Received	642	64.6	497	74.96	
Received	351	35.3	166	25.03	
Missing	278		115		9.16E-06

*Chemotherapy is adjuvant

Supplementary Table 5: Cross classification of visual and automated KI67 score categories

		Visual (25% cut-off)		
		**High**	**Low**	**Total**
**Automated (12% cut-off)**	**High**	417	295	712
	**low**	219	1,509	1728
	**Total**	636	1,804	2440
		**Sensitivity= 65.5**	**Specificity = 83.6**	

Supplementary Table 6: Multivariate model for the association of KI67 with 10-year BCSS among 5,520 ER positive patients

**Characteristic**	**HR**	**95% CI**	**P-value**
Low KI67	1.00 (ref)		
High KI67	1.96	1.31, 2.93	0.001
Age at diagnosis	1.01	0.99, 1.02	0.123
Grade 2 vs 1	1.65	1.30, 2.10	<0.0001
Grade 3 vs 1	2.77	2.12, 3.61	<0.0001
Size 2->5cm vs <2cm	1.51	1.26, 1.81	<0.0001
Size ≥ 5cm vs. <2cm	2.67	1.89, 3.76	<0.0001
Node positive vs negative	2.73	2.25, 3.30	<0.0001
PR positive vs negative	0.61	0.51, 0.73	<0.0001
HER2 positive vs negative	1.24	0.99, 1.54	0.055
Invasive lobular vs ductal	1.41	1.13, 1.75	0.003
Other morphology vs ductal	0.88	0.60, 1.28	0.494
Endocrine (yes vs no)	0.72	0.57, 0.90	0.004
Chemotherapy (yes vs no)	0.66	0.50, 0.87	0.003
T			
KI67	0.89	0.82, 0.96	0.006

Supplementary Table 7: Multivariate model for the association of KI67 with 10-year BCSS among 2,049 ER negative patients

Characteristic	HR	95% CI	P-value
Low KI67	1.00 (ref)		
High KI67	1.24	0.86, 1.77	0.248
Age at diagnosis	1.00	0.99, 1.01	0.758
Grade 2 vs 1	1.39	0.87, 2.21	0.165
Grade 3 vs 1	1.70	1.07, 2.69	0.024
Size 2->5cm vs <2cm	1.53	1.23, 1.90	<0.0001
Size ≥ 5cm vs. <2cm	2.38	1.60, 3.54	<0.0001
Node positive vs negative	3.59	2.87, 4.48	<0.0001
PR positive vs negative	0.47	0.34, 0.65	<0.0001
HER2 positive vs negative	1.37	1.10, 1.69	0.004
Invasive lobular vs ductal	1.06	0.70, 1.61	0.777
Other morphology vs ductal	0.86	0.56, 1.33	0.506
Endocrine (yes vs no)	1.01	0.77, 1.31	0.940
Chemotherapy (yes vs no)	0.60	0.44, 0.81	0.001
T			
KI67	0.95	0.87, 1.03	0.199
