# Supplementary figures and images for: Prognostic value of automated KI67 scoring in breast cancer: a centralised evaluation of 8088 patients from 10 study groups
Source: Breast Cancer Res. 2016 Oct 18;18:104. doi: 10.1186/s13058-016-0765-6 (PMC5070183; doi:10.1186/s13058-016-0765-6)

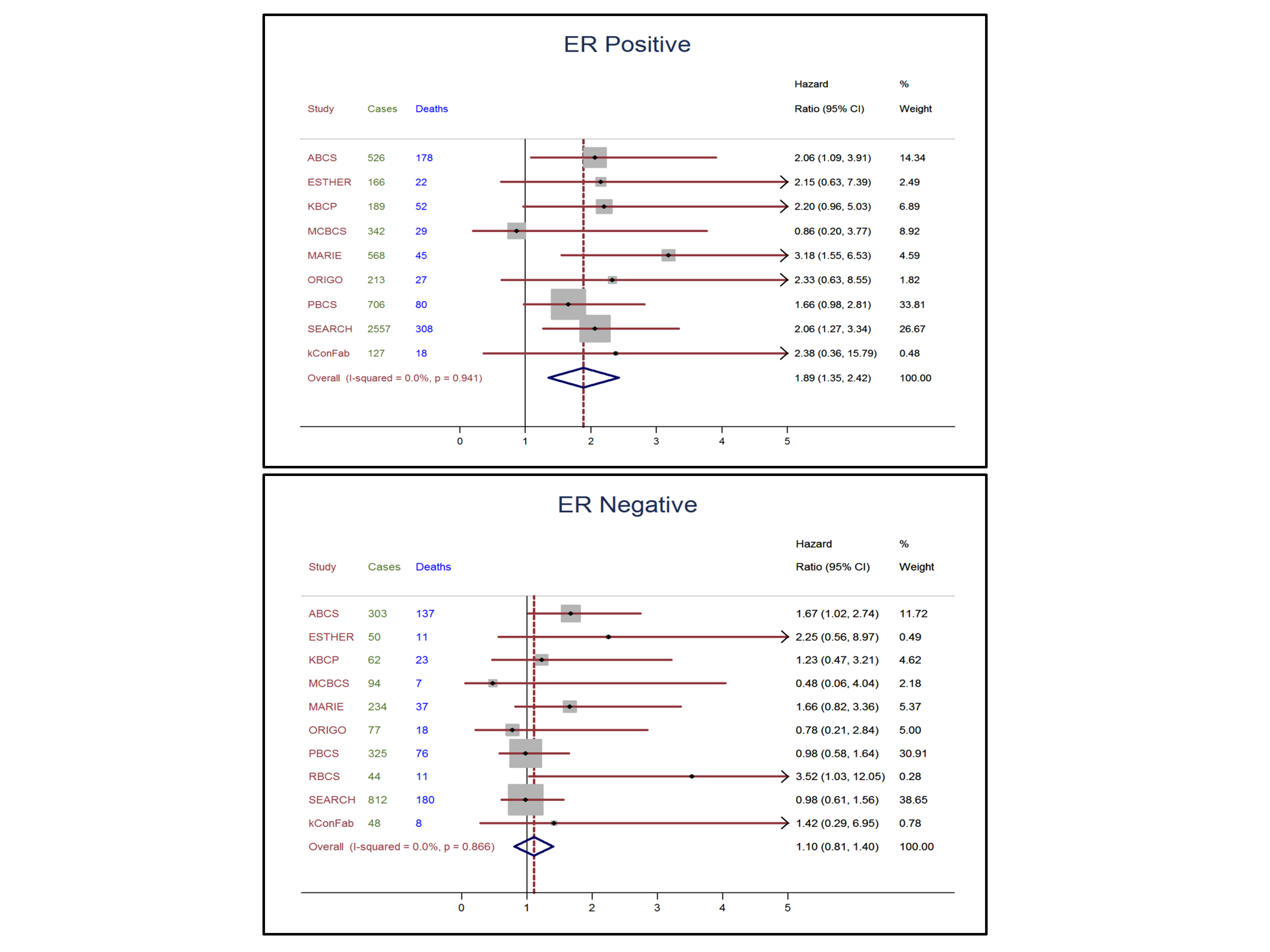

Supplement: Additional file 2: — Is Figure S1 showing meta-analysis of study-specific HR stratified by ER status. (TIF 1182 kb) [file 13058_2016_765_MOESM2_ESM.tif]

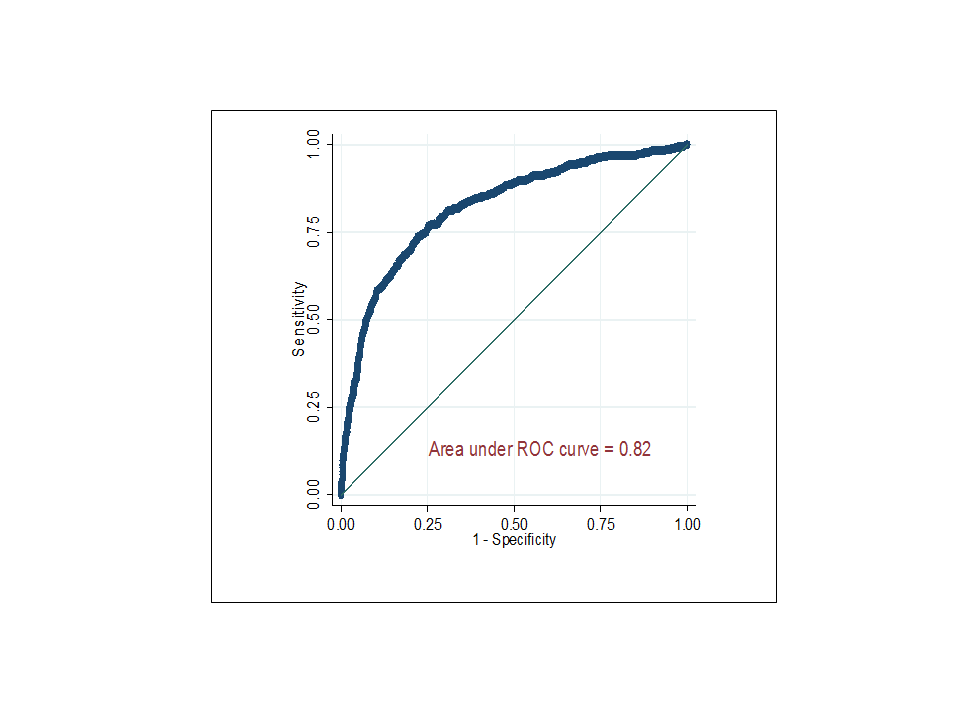

Supplement: Additional file 3: — Is Figure S2 showing ROC curve for the discriminatory accuracy of continuous automated KI67 scores against binary visual categories (≤25 % and > 25 %) based on a subset of patients with data on both visual and automated KI67 scores. (TIF 25 kb) [file 13058_2016_765_MOESM3_ESM.tif]

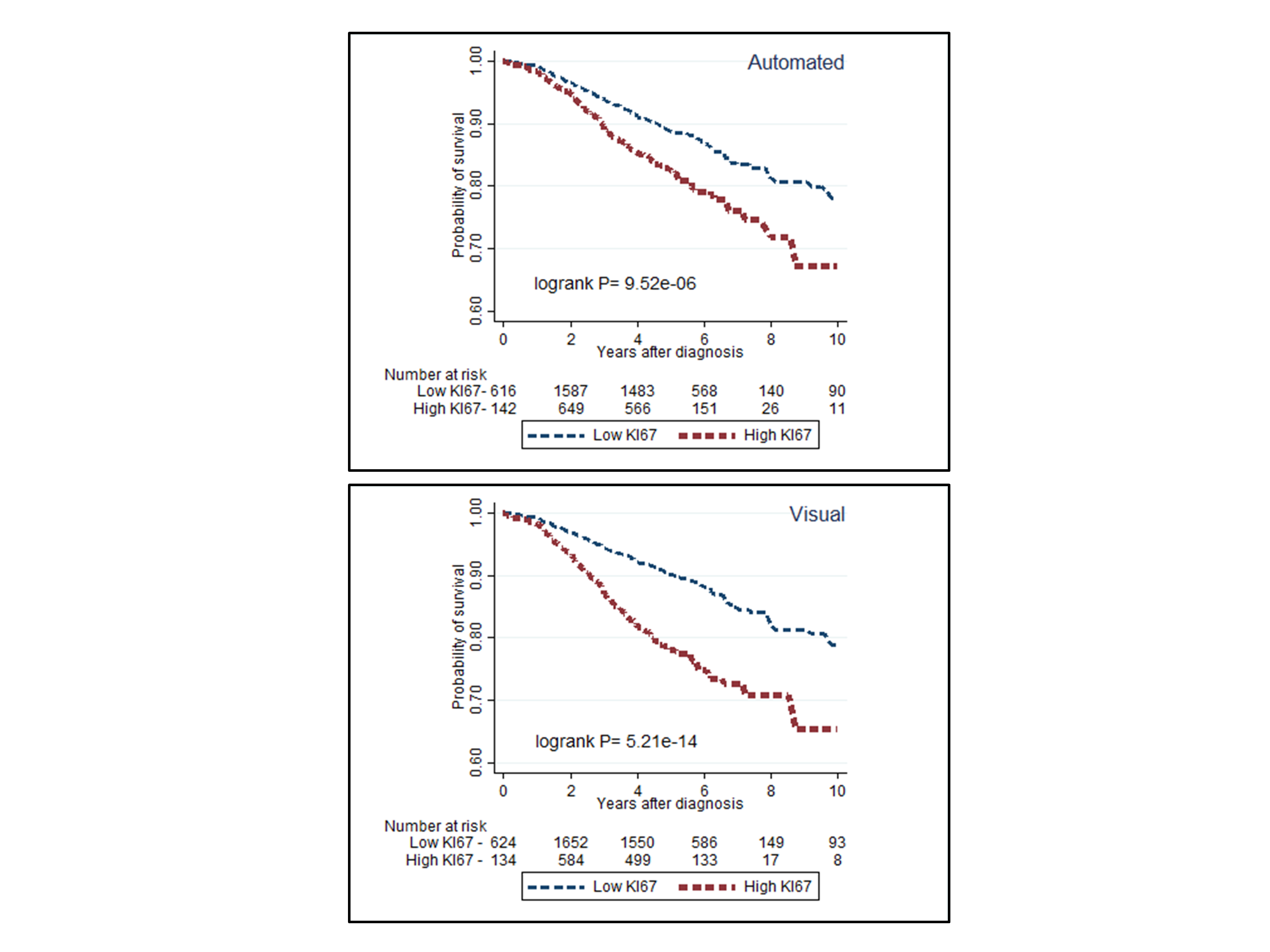

Supplement: Additional file 4: — Is Figure S3 showing Kaplan–Meier survival curves for the 10-year BCSS according to strata (high and low) of automated and visual KI67 scores (N = 2440). (TIF 1271 kb) [file 13058_2016_765_MOESM4_ESM.tif]
